# A Murine Model of *Mycobacterium kansasii* Infection Reproducing Necrotic Lung Pathology Reveals Considerable Heterogeneity in Virulence of Clinical Isolates

**DOI:** 10.3389/fmicb.2021.718477

**Published:** 2021-08-24

**Authors:** Vinicius O. Mussi, Thatiana L. B. V. Simão, Fabrício M. Almeida, Edson Machado, Luciana D. de Carvalho, Sanderson D. Calixto, Guilherme A. M. Sales, Eulógio C. Q. Carvalho, Sidra E. G. Vasconcellos, Marcos Catanho, Philip N. Suffys, Elena B. Lasunskaia

**Affiliations:** ^1^Laboratory of Biology of Recognition, State University of North Fluminense, Campos, Brazil; ^2^Laboratory of Molecular Biology Applied to Mycobacteria, Oswaldo Cruz Institute, Fiocruz, Rio de Janeiro, Brazil; ^3^National Reference Laboratory for Tuberculosis, Reference Center Professor Helio Fraga, National School of Public Health, Fiocruz, Rio de Janeiro, Brazil; ^4^Laboratory of Animal Morphology and Pathology, State University of North Fluminense, Campos, Brazil; ^5^Laboratory of Molecular Genetics of Microorganisms, Oswaldo Cruz Institute, Fiocruz, Rio de Janeiro, Brazil

**Keywords:** virulence, animal models, pulmonary disease, clinical isolates, *Mycobacterium kansasii*, nontuberculous mycobacteria, virulence factor genes

## Abstract

Among non-tuberculous mycobacteria, *Mycobacterium kansasii* is one of the most pathogenic, able to cause pulmonary disease indistinguishable from tuberculosis in immunocompetent susceptible adults. The lack of animal models that reproduce human-like lung disease, associated with the necrotic lung pathology, impairs studies of *M. kansasii* virulence and pathogenicity. In this study, we examined the ability of the C57BL/6 mice, intratracheally infected with highly virulent *M. kansasii* strains, to produce a chronic infection and necrotic lung pathology. As a first approach, we evaluated ten *M. kansasii* strains isolated from Brazilian patients with pulmonary disease and the reference strain *M. kansasii* ATCC 12478 for virulence-associated features in macrophages infected *in vitro*; five of these strains differing in virulence were selected for *in vivo* analysis. Highly virulent isolates induced progressive lung disease in mice, forming large encapsulated caseous granulomas in later stages (120–150 days post-infection), while the low-virulent strain was cleared from the lungs by day 40. Two strains demonstrated increased virulence, causing premature death in the infected animals. These data demonstrate that C57BL/6 mice are an excellent candidate to investigate the virulence of *M. kansasii* isolates. We observed considerable heterogeneity in the virulence profile of these strains, in which the presence of highly virulent strains allowed us to establish a clinically relevant animal model. Comparing public genomic data between Brazilian isolates and isolates from other geographic regions worldwide demonstrated that at least some of the highly pathogenic strains isolated in Brazil display remarkable genomic similarities with the ATCC strain 12478 isolated in the United States 70 years ago (less than 100 SNPs of difference), as well as with some recent European clinical isolates. These data suggest that few pathogenic clones have been widely spread within *M. kansasii* population around the world.

## Introduction

*Mycobacterium kansasii* is a slow-growing non-tuberculous mycobacterium (NTM) mainly found in aquatic environments that, differing from many other NTM, can cause human infections in both immunocompetent and immunocompromised individuals (Griffith et al., 2007). In susceptible immunocompetent individuals, the bacterium most frequently causes chronic pulmonary disease indistinguishable from tuberculosis (TB), resulting in the formation of cavities in more than 70% of cases, or, less frequently, in bronchiectasis or nodules; in contrast, in immunocompromised patients, the disseminated disease is most common (Seiscento et al., 2005; Matveychuk et al., 2012; Moon et al., 2015; Bakuła et al., 2018a; Goldenberg et al., 2020).

The prevalence of NTM pulmonary infections in humans have raised in recent decades, demonstrating a 2- to 8-fold increase in different geographic regions (Champa et al., 2020). The increase is thought to be associated with rapid growth of susceptible populations, driven by population aging, the HIV epidemic and increase in number of individuals with alterations of lung health, as a result of air pollution, smoking, an increase in prevalence of immune-modulating comorbidities like diabetes mellitus, immunosuppressive medication use, as well as sequelae of previous TB and other lung diseases, such as obstructive pulmonary disease (Falkinham, 1996). In the nearest future, susceptibility to *M. kansasii* and other NTM infections may increase due to the widespread emergence of post-COVID-19 syndrome associated with pulmonary sequelae (Nalbandian et al., 2021).

Among human pathogenic NTM species, *M. kansasii* is one of the most prevalent, only surpassed by *Mycobacterium avium* complex in most countries (Hoefsloot et al., 2013). However, in some geographic regions, such as Central Europe or Rio de Janeiro in Brazil, *M. kansasii* is the most common NTM isolate in patients with pulmonary disease, raising the questions about specific features of the local strains, population or transmission (de Mello et al., 2013).

Genetic studies of clinical and environmental *M. kansasii* isolates (McSwiggan and Collins, 1974; Steadham, 1980; Taillard et al., 2003) have demonstrated that *M. kansasii* is a heterogeneous species divided into six genetically distinct subtypes (I–VI) by genotyping (Bakuła et al., 2018b). Recent comparative genomic analyses revealed that these subtypes are more accurately represented as closely related subspecies: *Mycobacterium kansasii* (I), *Mycobacterium persicum* (II), *Mycobacterium pseudokansasii* (III), *Mycobacterium ostraviense* (IV), *Mycobacterium innocens* (V) and *Mycobacterium attenuatum* (VI); together with *Mycobacterium gastri* these species form the *M. kansasii* complex (Jagielski et al., 2020). The clinical isolates obtained from patients with pulmonary disease in distinct geographic regions of the globe are almost exclusively composed by subtype I strains (Alcaide et al., 1997; Taillard et al., 2003; Chimara et al., 2004), currently considered *M. kansasii* in the strict sense (Jagielski et al., 2020), suggesting that they are better adapted to the host and display higher virulence.

Research on virulence and pathogenicity of *M. kansasii* isolates is constrained by the lack of reliable animal models of *M. kansasii* infection reproducing chronic infection and human-like pathology. Evaluation of virulence-associated features commonly applied to pathogenic mycobacteria, such as the survival fitness and the cytotoxicity of mycobacteria in macrophage culture infected *in vitro*, was proposed for initial screening of virulence profile of *Mycobacterium tuberculosis* (Lasunskaia et al., 2010) and *M. kansasii* (Sohn et al., 2010). However, a complete range evaluation of host-pathogen interactions determining disease development needs an appropriate animal model of infection.

*Mycobacterium kansasii* is generally less virulent than *M. tuberculosis*, and therefore high doses of bacilli (10^6^ or 10^7^ bacilli/mouse) were used in previous studies for intravenous or subcutaneous injection into immunodeficient beige or athymic mice (Graybill and Bocanegra, 2001; Cynamon et al., 2003) or immunocompetent mice of different strains (C57BL/6, B6D2F1, and CD-1) to avoid rapid clearance of mycobacteria and induce a sustained infection (Collins et al., 1975; Hepper and Collins, 1984; Flory et al., 1992). More recently, lower doses (10^5^ bacilli/mouse) of the *M. kansasii* ATCC 12478 strain or of clinical isolates were applied for infection via intranasal or aerosol routes in the C57BL/6 mice model; yet this model was only able to reproduce a non-replicative or low replicative infection (Wieland et al., 2006; Wang et al., 2015; Ghanem et al., 2020). However, none of the described models reproduced the features of chronically progressive infection and necrotic lung pathology (caseous granulomas or cavities), typical of *M. kansasii* disease in humans.

Considering the role of the virulence of *M. tuberculosis* isolates in the severity of experimental TB (Ribeiro et al., 2014), we anticipated that the *M. kansasii* clinical isolates, exhibiting a higher degree of virulence compared to the reference strain 12478, might induce more severe lung pathology and disease in C57BL/6 mice. To verify this hypothesis, we first tested the virulence–associated features of *M. kansasii* strains from our collection of genetically well-characterized clinical isolates (Machado et al., 2018), employing the model of macrophage infection *in vitro*, then selecting five strains with different degrees of predicted virulence (high, intermediate, and low) for studies in mice. Our results demonstrate that isolates exhibiting high or intermediate degrees of *in vitro* virulence, when intratracheally inoculated at a relatively low dose of 5.0 × 10^4^ bacilli/mouse, could induce progressive lung disease, resulting in formation of large encapsulated caseous granulomas in 4–5 months post-infection; in contrast, the low-virulence strain was eliminated from the lungs in 40 days. Hence, resistant C57BL/6 mice infected with a low dose of virulent *M. kansasii* reliably reproduce the human-like lung disease typically caused by these bacteria.

## Materials and Methods

### Bacterial Culture

In the present study, we analyzed eleven strains of *M. kansasii*: the reference strain ATCC 12478 and ten clinical isolates obtained from Brazilian patients with pulmonary disease (Machado et al., 2018). The diagnosis of pulmonary disease caused by NTM was performed generally following the Brazilian Ministry of Health guidelines and an official statement by the American Thoracic Society/Infectious Diseases Society of America (Griffith et al., 2007), based on respiratory symptoms, image findings (e.g., nodular or cavitary opacities on X-rays or multifocal bronchiectasis and multiple small nodules), and positive culture for NTM in sputum or BAL fluid specimens, or a positive tissue culture following lung biopsy. The bacteria were grown in Middlebrook 7H9 medium (BD Difco, MD) supplemented with 0.05% glycerol and 10% ADC (albumin-dextrose-catalase enrichment – BD BBL) at 37°C. After 5 days of cultivation, bacterial suspensions were treated in an ultrasonic bath at 42 kHz, vortexed, and kept for 10 min for sedimentation of eventual clumps. The optical densities (O.D.) of the resulted suspensions were measured by spectrophotometry at a wavelength of 600 nm, adjusted to an OD_600_ of 0.2; the corresponding bacterial concentrations were determined by serial dilution and plating of bacteria on Middlebrook 7H10 agar (Difco, Detroit, MI) plates, supplemented with 0.05% glycerol and 10% OADC (oleic acid–albumin-dextrose–catalase enrichment – BD, Sparks, MD).

All bacterial strains were grown on Middlebrook 7H10 agar with supplements for 3–4 weeks to determine colony morphotype. Colonies were examined with a stereomicroscope (×4 to ×20 magnification; model 569; American Optical Corp., Buffalo, NY) and an external light source. Images were captured with a Samsung Zoom Lens camera. The *M. kansasii* colonies displayed either rough or smooth polar morphology, as expected.

### Macrophage Culture Infection

Murine macrophage-like cells RAW 264.7 (ATCC, VA, United States) were cultured in Dulbecco’s modified Eagle medium–nutrient mixture F-12 (DMEM-F12), complemented with 10% fetal bovine serum (FBS) and 50 μg/mL gentamicin (Gibco BRL, Grand Island, NY), at 37°C and 5% CO_2_.

Cells were resuspended in a complete DMEM-F12 medium supplemented with 2% FBS without antibiotics (5 × 10^5^ cells/mL) and plated on 96-well plates (0.1 mL/well) for cell adherence and formation of a monolayer. After 24 h incubation at 37°C, cells were infected with each strain for three hours. Then, the extracellular non-phagocytosed bacteria were removed by washing with PBS. Subsequently, fresh aliquots of the medium were added to the infected cultures. The cells were incubated at 37°C for 3 or 4 days, and then examined for bacterial growth and macrophage death.

### *In vitro* Quantification of Intracellular Mycobacterial Growth

Macrophage cultures were infected with each strain at a multiplicity of infection (MOI) of 1:1 (bacteria/macrophage). On the first and fourth day post-infection (p.i.), cells were lysed with 0.1% saponin solution and 20 min of incubation. The culture lysate was collected, treated in an ultrasonic bath and vortexed to break up any bacterial clumps, serially diluted in PBS, and submitted to the CFU (colony-forming unit) test. For this, 50 μL aliquots of each dilution were seeded on Middlebrook 7H10 agar. The plates were incubated for 3 weeks at 37°C and colonies were quantified. Bacterial counting was expressed in log_10_; intracellular growth was quantified, subtracting the average CFU obtained from macrophages on day 4 and day 1. Growth values of each isolate were calculated as a proportion of the reference strain *M. kansasii* ATCC 12478 growth value set as 1.0 for comparison (index A).

### *In vitro* Evaluation of Cell Death Caused by Mycobacteria

Macrophage cultures were infected with each strain at a MOI of 10:1 and cultured for 3 days. Induction of necrotic cell death in infected cultures was evaluated, measuring the release of lactate dehydrogenase (LDH) enzyme into the culture supernatant, using the Labrax commercial Kit (GO, Brazil). Supernatants were collected on day 3 p.i. and assayed according to the manufacturer’s instructions. The O.D. was measured with a plate spectrophotometer at wavelength of 492 nm (Dinatech MR5000). Macrophage cultures treated with 1% (v/v) Triton X-100 (Sigma Aldrich) were used as a positive control for maximum LDH release. Spectrophotometric measurements were corrected, subtracting the value obtained with the culture medium alone from the untreated (non-infected), test (infected), and positive control samples. The percentage of cytotoxicity was calculated using the corrected O.D. values and following formula: Cytotoxicity (%) = (test – untreated)/(maximum LDH release control – untreated) × 100. Relative cytotoxicity of each isolate was calculated as a proportion of the reference strain *M. kansasii* ATCC 12478 cytotoxicity value set as 1.0 for comparison (index B). Relative virulence index was calculated for each isolate as an average of two values [(index A + index B)/2].

### Mice Infection

Specific-pathogen-free C57BL/6 male mice, aged between 6 and 8 weeks, were acquired from the State University of North Fluminense (UENF) bioterium and transferred to an animal biosafety level 3 facility before infection. All experimental procedures were approved by the Institutional Animal Care and Use Committee (Permit number 350/2017). Mice were infected intratracheally (i.t.), as described previously (Ribeiro et al., 2014), with 5.0 × 10^4^ CFU/mouse, as this dose previously was demonstrated to induce persistent *M. kansasii* lung infection in C57BL/6 mice (Wieland et al., 2006). The inoculum dose was confirmed by a CFU count of mouse lung tissue homogenates obtained 24 h p.i. Disease progression was monitored, weighing before the challenge and then every 7 days. Signs of illness or impairment, such as a reduced food intake, lack of mobility, alterations in hair coat and respiratory difficulty, were noted daily. A weight loss exceeding 25% of the initial body weight, combined with at least some of the clinical signs of disease, was an indication for applying euthanasia to minimize the suffering of the moribund animals according to humanitarian endpoint guidelines. Data collected from the moribund mice that underwent euthanasia or died spontaneously were used to calculate the survival proportion, using the Kaplan-Meier test.

Following the disease monitoring, animals infected with different *M. kansasii* strains (12 animals/group), were sacrificed and lungs were harvested at 28, 40, 60, 120, and 150 days after infection to evaluate mycobacterial growth and pathological alterations in the lungs.

The lung lobes were washed with sterile PBS and weighed. The lung relative mass was calculated as a fraction of the average lung weight of each experimental group and the average lung weight of uninfected controls. For bacterial counts, the left lung was homogenized, and serial dilutions of the homogenate were plated on complete Middlebrook 7H10 agar for the CFU test.

### Lung Pathology

Right lungs were fixed in 10% buffered formaldehyde. The upper lobes of each animal were photographed, and subsequently, lungs were embedded in paraffin. For histopathological studies, serial 4- to 5-μm sections were stained with hematoxylin and eosin (H&E) to visualize tissue alterations, with the Ziehl-Neelsen method to detect the presence of acid-fast bacteria (AFB), and Masson’s trichrome staining method to visualize collagen fibers. Samples were examined with an Axioplan 2 microscope (Carl Zeiss, Germany), and images of lung sections of at least four mice per group were captured by Axiocam MCR-5 (Zeiss)-coupled device camera.

For morphometric analysis, images were obtained at a magnification of 10×, and the Image J program (NIH, Bethesda, MD) was used to assess the area of inflammation (non-aerated area). Color images were converted to a black and white scale to allow software identification of aerated areas, such as the alveoli (in black) and non-aerated areas, including inflamed and non-inflamed tissue (in white). The average percentage of white area (non-aerated tissue) for 10 lung sections of control uninfected mice, and each of the different infected groups, was determined. To quantify the percentage of the inflamed area (area of pneumonia) in the lungs of infected animals, the average percentage of the non-aerated tissue area of control mice was subtracted from the average percentage of the non-aerated areas in each infected group.

### Virulence Factor Genes of *Mycobacterium tuberculosis* H37Rv and Homologous Counterparts in *Mycobacterium kansasii* ATCC 12478 and Clinical Isolates

*Mycobacterium tuberculosis* H37Rv genes (GenBank accession AL123456.3) with putative virulence activity were obtained from the literature (Chen et al., 2016; Mikheecheva et al., 2017). Functional category assignment of VFs follows the classification implemented in Mycobrowser (Kapopoulou et al., 2011). Orthology assessment analysis was performed with OMA standalone version 2.3.1 (Altenhoff et al., 2019), employing default parameters, to uncover VFs homologous genes shared between *M. tuberculosis* H37Rv and *M. kansasii* ATCC 12478. Variant calling analysis of *M. kansasii* clinical isolates genomes was performed with Snippy version 3.2^1^, employing default parameters and *M*. *kansasii* ATCC 12478 (GenBank accession CP006835.1) as reference.

### Statistical Analysis

Statistical analyzes were performed using the GraphPad Prism 4 software (GraphPad, United States); differences between the experimental groups were considered significant when *p* < 0.05 (5%). A one-way ANOVA test followed by Tukey’s multiple comparison test was applied to assess the effects of a single parameter when comparing multiple groups. Survival curves were analyzed with the log-rank test of the Kaplan-Meier method.

## Results

### Colony Morphotype, Intracellular Growth and Macrophage Death Induction by *Mycobacterium kansasii* Isolates

**FIGURE 1:**
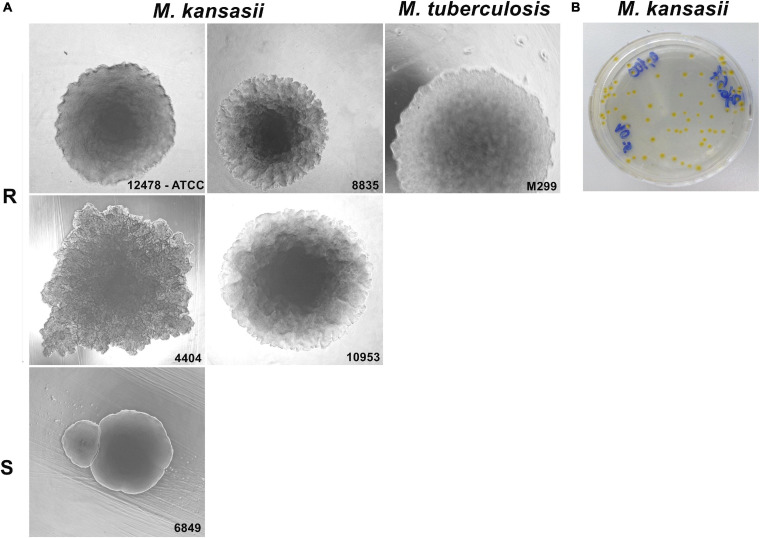
Colony morphotype variants of *Mycobacterium kansasii* strains. Mycobacterial strains were grown on Middlebrook 7H10 agar for 21 days (*M. kansasii*) and 28 days (*Mycobacterium tuberculosis*), and the macrocolony images were captured. **(A)** *M. kansasii* clinical isolates (images are demonstrated for strains 8835, 4404, and 10953) exhibited a rough colony shape (R) similar to that of the reference *M. kansasii* strain 12478 or the *M. tuberculosis* strain H37Rv, with exception of the isolate 6849, which showed smooth morphotype (S). **(B)** All *M. kansasii* strains grown on the agar exhibited yellow colonies under exposure to light.

Colony morphology of ten clinical isolates of *M. kansasii* was compared with that of the reference ATCC 12478 strain (Figure 1). Nine of the ten isolates exhibited rough circular colonies with a dry texture and undulated margins, as the reference *M. kansasii* strain 12478 (R strains), except isolate 6849 exhibiting smooth and moist colonies (S strain). The shape of the R strains resembled that of the *M. tuberculosis* strain H37Rv (Figure 1A); however, in contrast to the cream-colored colonies exhibited by *M. tuberculosis*, all *M. kansasii* isolates displayed photochromogenic capacity, producing yellow pigment under exposure to light (Figure 1B).

**FIGURE 2:**
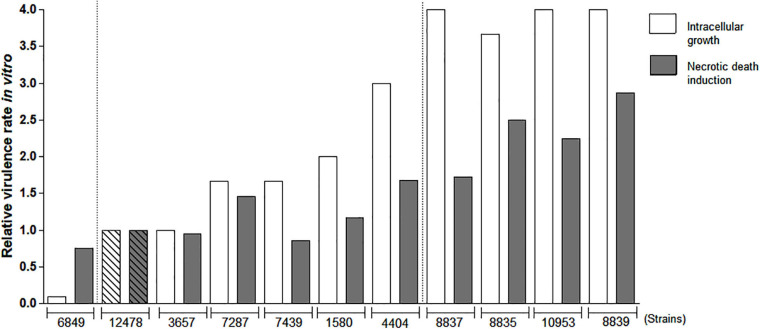
Evaluation of relative virulence of *Mycobacterium kansasii* strains as measured by *in vitro* macrophage infection. Mycobacterial ability to intracellular growth in macrophages and induction of macrophage death was evaluated to calculate relative virulence of each *M. kansasii* isolate compared with that of the reference virulent *M. kansasii* strain 12478. RAW264.7 macrophage-like cells were infected with the studied strains at a multiplicity of infection (MOI) of 1:1 and cultured for 4 days. Intracellular bacterial growth was evaluated by CFU test. The mean growth value of reference strain 12478 was established as 1.0 and relative growth rate of each clinical isolate was calculated proportionally. To evaluate macrophage death, the cells were infected at a MOI of 10:1, cultured for 3 days, and necrotic cell death was evaluated by quantification of lactate dehydrogenase (LDH) release (LDH test). Relative cytotoxicity of each isolate in relation to the cytotoxicity of reference strain 12478 (established as 1.0) was calculated proportionally.

Since intracellular growth and macrophage death induction were previously associated with virulence in pathogenic mycobacteria (Lasunskaia et al., 2010; Sohn et al., 2010), we evaluated these features in *M. kansasii* clinical isolates. Relative values of growth and cytotoxicity of each isolate compared to the reference strain ATCC 12478 were calculated based on the data of mycobacterial intracellular growth and cytotoxicity in macrophages (Supplementary Figure 1). These data demonstrated that a proportion of isolates (4404, 8835, 8837, 8839, and 10953) presented higher capacity to grow and induce death in macrophages comparatively to the reference strain, while the remaining isolates displayed either similar (1580, 3657, 7287, and 7439) or lower (6849) capacities (Figure 2). The average of these two values [(index A + index B)/2] was calculated for each isolate and is presented in the Supplementary Table 1 as a relative virulence index. Strains exhibiting at least 2.5-fold higher or 2.5-fold lower relative virulence index than that of the reference strain were considered as high- or low-virulence strains, respectively. According to these *in vitro* data, only one strain (isolate 6849) was less virulent than the strain 12478, at least four isolates (8835, 8837, 8839, and 10953) exhibited increased virulence and remaining isolates, including the reference strain 12478, were moderately virulent (Supplementary Table 1).

### Evaluation of *Mycobacterium kansasii* Virulence in the Model of C57BL/6 Mice Infection

**FIGURE 3:**
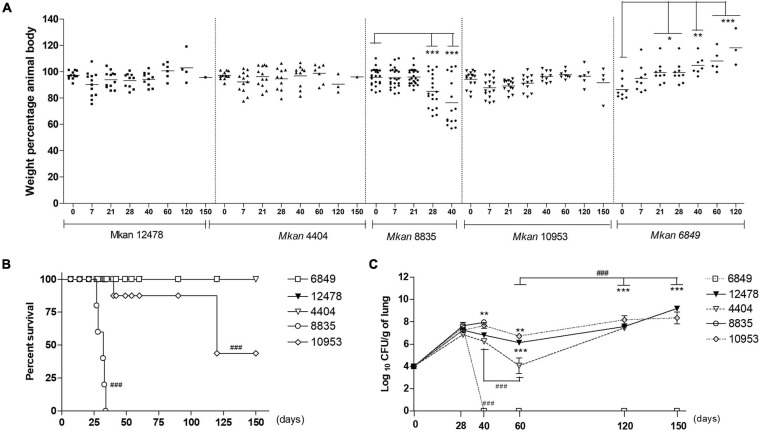
Morbidity and mortality of mice after challenge with *Mycobacterium kansasii* strains. C57BL/6 mice were infected intratracheally with 5 × 10^4^ bacilli of each strain and the disease progression was studied within a 150-day period. **(A)** Body weight loss was used as an indicator of morbidity. Data are presented as the percentage of initial body weight of each animal prior to infection. Values were reported as mean ± standard deviation, SD, and the differences were considered significant according to ***P < 0.001; **P < 0.01; and *P < 0.05. **(B)** Survival of the infected animals. The data were obtained in three independent experiments with 10 mice in each group. Kaplan-Meier curves and log rank test were used to evaluate statistical significance. Statistically significant differences between each group infected with the individual clinical isolate and the group infected with reference strain 12478 are presented by symbol ^###^*P* < 0.001. **(C)** Bacterial loads in the lungs were assessed by CFU assay. The data were obtained in three independent experiments with 12–15 mice in each group: 2–3 animals per point. Values were reported as mean ± SD. Mean values that were significantly different from the mean value of the group infected by reference strain 12478 are indicated by asterisks as follows: ***P < 0.001 and **P < 0.01. Significant differences between values obtained for each group at different time points are indicated by symbol ^###^*P* < 0.001.

Based on the results of *in vitro* testing, we selected five isolates for virulence tests in an animal model of mycobacterial infection: two intermediate virulent strains (pattern II strains ATCC 12478 and 4404), one low virulent strain (pattern III strain 6849) and two high virulent strains (pattern I strains 8835 and 10953). The infected animals were weighed weekly to verify the body weight change, and weight loss was used as an indicator of morbidity. Mice infected with the less virulent strain 6849 presented body weight gain starting at day 40 p.i., while animals in other infected groups either maintained the body weight values unaltered or exhibited significant weight loss (strain 8835), starting at day 28 (Figure 3A). The survival analyses demonstrated the premature death of mice in group 8835 at day 40 p.i., and 60% lethality in group 10953 at day 120 p.i., while in the remaining groups, animals survived until the end of the experiment (Figure 3B).

The analysis of pulmonary bacterial loads demonstrated that all strains, including the less virulent strain 6848, could grow in the lungs up to day 28 p.i. (Figure 3C). The most virulent strain 8835 induced higher bacterial loads (log_10_ 8.0) by the day 40 p.i., coinciding with deterioration of the animals in this group that reached a moribund state and were euthanized. In contrast, the pulmonary bacterial loads in other groups started to drop after day 28, demonstrating only a slight reduction in mice infected with strains 10953 and 12478, and more than a 2-log reduction in group 4404, at day 60, while in mice infected with low virulent strain 6849, bacteria were eliminated from lungs by the day 40 p.i. In groups comprising animals developing chronic infection, the bacterial loads in the lungs started to increase again achieving log_10_ 8.0–9.0 level on day 150 p.i.

**FIGURE 4:**
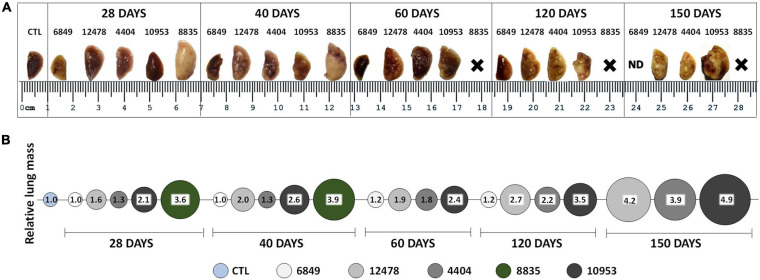
Macropathological changes in the lungs of mice infected with *Mycobacterium kansasii* strains. C57BL/6 mice were infected as indicated in the Figure 3 legend, and lungs were examined on day 0, 28, 40, 60, 120, and 150 p.i. **(A)** Representative images of the upper lobe of the right lung demonstrating the gross pathology in animals of each infected group, observed as numerous giant inflammatory lesions (white nodes of different size). **(B)** Relative lung mass in different infected groups was determined by the ratio of the mean lung weight of animals in each group to the mean lung weight of control (CTL) animals (1.0). ND, not demonstrated; X-premature animal death.

**FIGURE 5:**
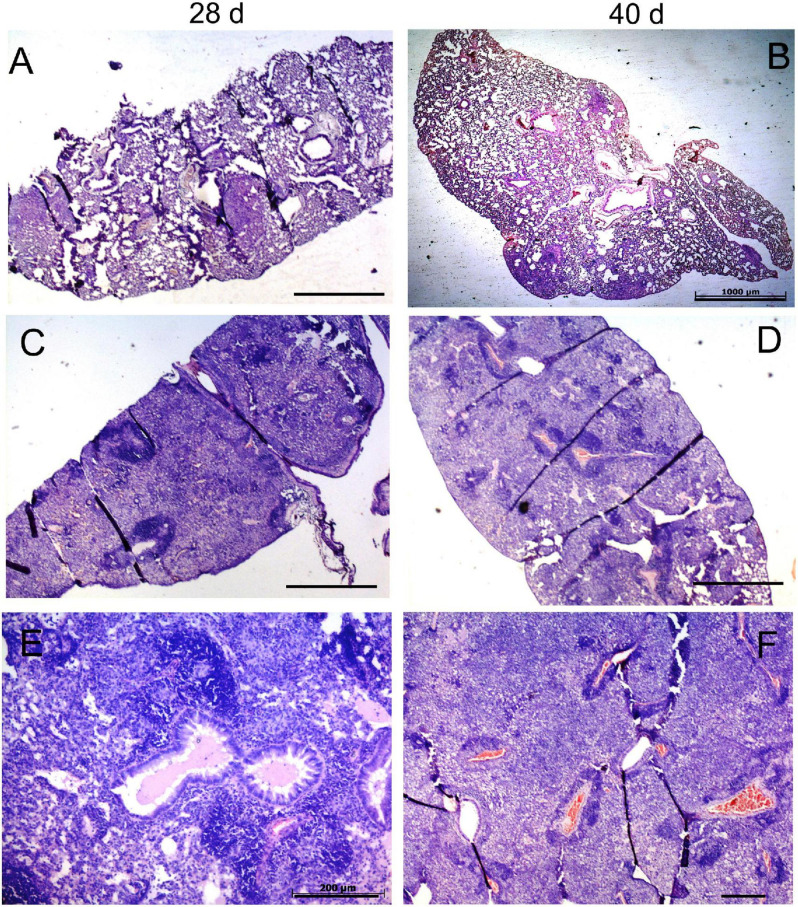
Histopathological changes in the lungs of mice infected with low-virulence strain 6849 and high-virulence strain 8835. C57BL/6 mice were infected with strain 6849 **(A,B)** or strain 8835 **(C–F)**, and lungs were examined on day 28 **(A,C,E)** and day 40 p. i. **(B,D,F)**. Representative hematoxylin-and-eosin (H&E) -stained lung sections. Few small-size granulomas **(A,B)**. Extensive pneumonia, leading to consolidation of lung tissue **(C–F)**. Note accumulation of exudates in the respiratory bronchioles on day 28 p.i. **(E)**, and blood coagulation in the vessels and diffuse necrotic cell death in the lung parenchima on day 40 p.i. **(D,F)**. Bars, 1,000 μm **(A–D)**, 200 μm **(E,F)**.

**FIGURE 6:**
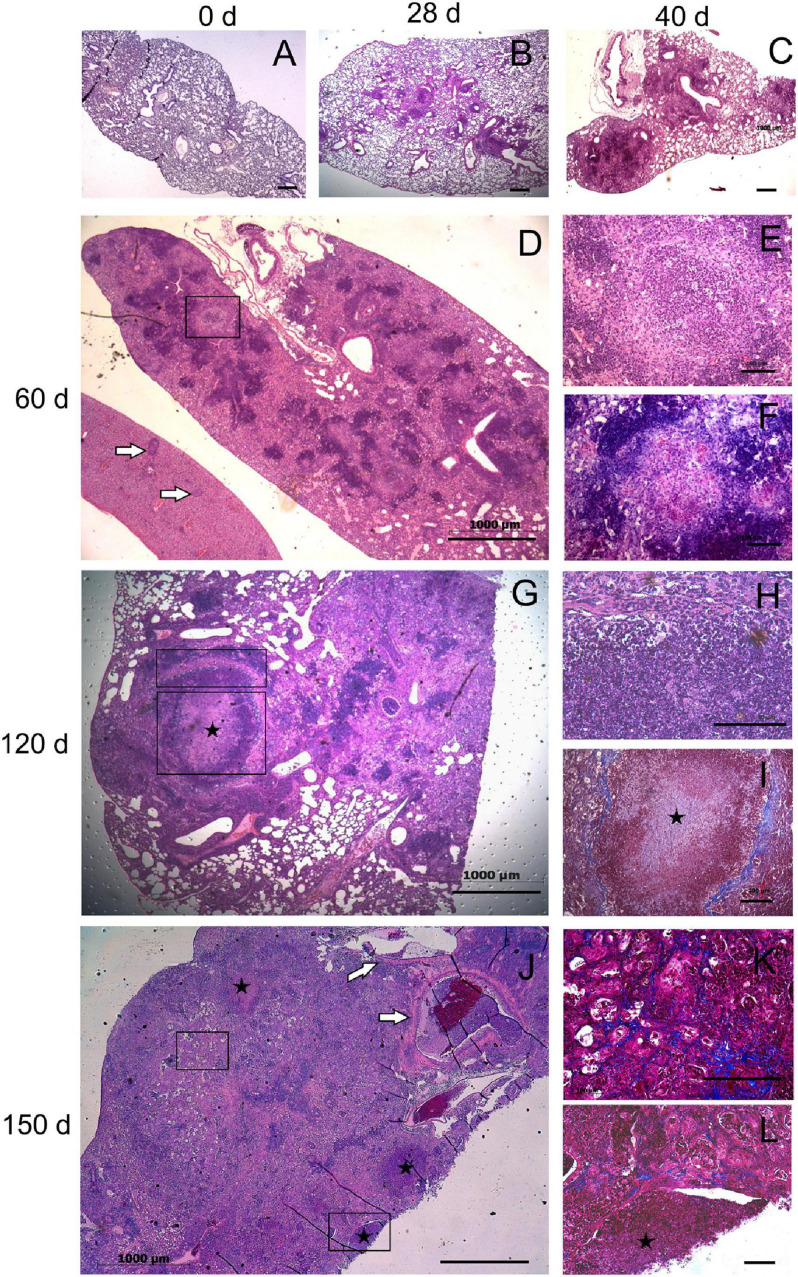
Histopathological changes in the lungs of mice infected with highly virulent *Mycobacterium kansasii* strain 10953. C57BL/6 mice were infected, and lung pathology was examined during a 150-day period, on day 0 **(A)**, 28 **(B)**, 40 **(C)**, 60 **(D–F)**, 120 **(G–I)** and 150 **(J–L)**. Representative lung sections stained by H&E **(A–E,G,H,J)**, Ziehl-Neelsen **(F)** or Masson’s Trichrome methods **(I,K,L)** are shown. Small granulomas seen on day 28 **(B)**, increased by day 40 **(C)**, and progressed to extensive granulomatous pneumonia by day 60 **(D)**. Area of initial intragranulomatose necrosis, marked by black square in panel **(D)**, is enlarged in panels **(E,F)**, demonstrating large numbers of dying cells **(E)** and AFB **(F)**. In panel **(D)**, a liver section **(l)** eventually is localized by side of the lung section; white arrows indicate several granulomas in the liver. By day 120, necrotic lesions progressed to large caseous granulomas marked by black squares **(G)**. The upper and the lower squares refer to areas of higher magnification in panel **(H)** (peripheral area composed by neutrophil-predominant cell infiltrates) and **(I)** (central area occupied by acellular caseum), respectively. Note a fibrotic capsule surrounding the necrotic lesion **(I)**. By day 150 **(J)**, necrosis leads to destruction of normal lung structure (symbol *) with easy dislocation of the amorphous necrotic material [lower black square enlarged in panel **(L)**] and its presence in occluded bronchial airways [black arrows in panel **(J)**]. The area marked by upper square in panel **(J)**, enlarged in panel **(K)**, demonstrates significant interstitial fibrosis of the lung. Bars, 1,000 μm **(A–D,G,J)**, 100 μm **(E,F,H,I,K)**.

We monitored the gross pathology (Figure 4) and histological changes (Figures 5, 6) in the lungs for 150 days p.i. to evaluate *M. kansasii*-induced pathology progression. On day 28 p.i., uniformly distributed small white nodes were observed in the lungs of all infected animals, except mice infected with the highly virulent strain 8835, which exhibited a diffuse lobular consolidation instead (Figure 4A). In this group, animals exhibited signs of severe illness and were euthanized at day 40 p.i. Lung lesions in other groups either continued to expand and finally fused, forming large well-demarcated nodules at the later stages of infection, or continued without significant morphological changes (group 6849). On day 150 p.i., the pathological nodules in the lungs of some animals infected with strain 10953 presented ulcerated surface alterations, suggesting pleural effusion (Figure 4A).

Three different scenarios of histopathology development in the lungs were observed in animals of different infection groups. Pathological lung lesions in mice infected with the strain 8835 developed faster than in other groups and were characterized by extensive granulomatous inflammation, leading to lobular pneumonia at day 28 p.i., with almost complete consolidation of the lung tissue at day 40 (Figures 5C–F). In contrast, the low virulent strain 6849 induced a few small granulomas at day 28 p.i., while further development of these lung lesions was aborted (Figures 5A,B). The third scenario of the development of lung lesions, characterized by a permanent slow progression of the initial granulomas, was observed in animals infected with strains 12478, 4404, and 10953. Histopathological changes in the lungs of these animals are demonstrated for strain 10953, as representative for these three infected groups (Figure 6). In these animals, small initial granulomas increased gradually and, at day 60, various foci of intragranulomatous necrosis (Figures 6D–F), composed of dying macrophages, and numerous extracellular AFB, surrounded by a collar of lymphocytes, were observed. Further progression led to large circumvented caseous granulomas surrounded by a fibrous capsule, with large numbers of neutrophils in sub capsular regions and the central area occupied by acellular caseum (Figures 6G–I). On day 150 p.i. (Figure 6J–L), tissue necrosis led to the destruction of typical lung structure, with easy dislocation of the amorphous necrotic material (lower black square), occlusion of bronchial airways (black arrows in J), and pleural effusion. Significant interstitial fibrosis with many foamy cells contributed to the consolidation of the surrounding lung tissue. Expansion of lesions in animals infected with strains 12478 and 4404 was somewhat delayed compared with those infected with strain 10953. Development of the caseous granulomas in former groups was only found after 3 months of infection (150 days p.i.), coinciding with better survival of the animals.

**FIGURE 7:**
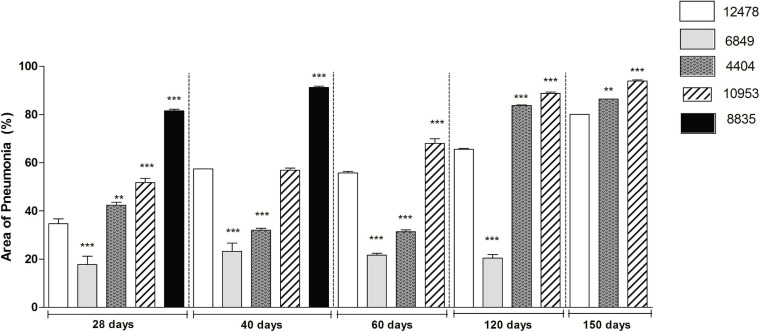
Morphometric analysis of lung pathology in animals infected with *Mycobacterium kansasii* strains. For morphometric determination of the inflammatory area occupied in the lungs by cellular or liquid exudates (pneumonia area), images of ten lung sections of each group of animals were captured and analyzed using the Image J program. Statistically significant differences between each group infected with the individual clinical isolate and the respective group infected with reference strain 12478 are presented by asterisks as follows: ***P < 0.001 and **P < 0.01.

Morphometric analysis of the area occupied by pneumonia demonstrated that the infection with most virulent *M. kansasii* strains (8835 and 10953) led to impairment of 90% of lung tissue at the final pre-lethal stage of infection, although the duration of the disease before death varied according to the virulence of the causing bacteria (Figure 7).

### Virulence Factors and Genetic Variations

We found 299 orthologous counterparts of the 457 *M. tuberculosis* H37Rv virulence factor (VF) genes (Chen et al., 2016; Mikheecheva et al., 2017) encoded in *M. kansasii* ATCC 12478 genome (Supplementary Table 2).

**FIGURE 8:**
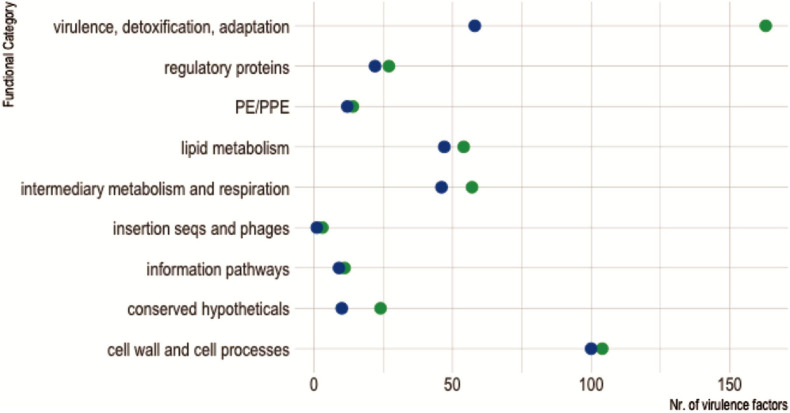
Virulence factors shared between *Mycobacterium kansasii* ATCC 12478 and *Mycobacterium tuberculosis* H37Rv, according to their functional categories. The diagram shows the distribution of virulence factors encoded in each mycobacterial genome according to their assigned functional category, as defined in Mycobrowser classification (https://mycobrowser.epfl.ch/). Green dots represent *M. tuberculosis* H37Rv virulence factors, while blue dots account for homologous counterparts in *M. kansasii* ATCC 12478.

As expected, since *M. kansasii* expresses less VF and is less virulent than *M. tuberculosis*, we observed that genes encoding VFs belonging to the functional category “virulence, detoxification, adaptation” had undergone a massive reduction in *M. kansasii* compared with *M. tuberculosis* H37Rv (Figure 8).

An interesting observation is that *M. tuberculosis* H37Rv genes Rv1969/*mce3D*, Rv1970/*lprM*, and Rv1971/*mce3F* (Mce protein family), as well as genes Rv1982A/*vapB36* and Rv1982c/*vapC36* (anti-toxin type 2 system), have two homologous copies encoded in *M. kansasii* ATCC 12478 genome in which one copy is inferred to be chromosomal (Mce protein family loci MKAN_23035, MKAN_23040, and MKAN_23045; anti-toxin type 2 system loci MKAN_23045, MKAN_05465 and MKAN_05470, respectively), while the second copy is predicted to be encoded in plasmid pMK12478 (loci MKAN_29245, MKAN_29250, and MKAN_29255; loci MKAN_29500 and MKAN_29505, respectively) (Supplementary Table 2).

To seek genetic variations in VF genes that could correlate with the broad virulence spectrum observed in *M. kansasii*, we performed a single nucleotide polymorphism (SNP) analysis, comparing the reference strain ATCC 12478 with the Brazilian strains (Supplementary Table 3). Identical SNPs in gene MKAN_07615, encoding for ESX secretion-associated protein EspG, were found in the strains 1580, 3657, 4404, 8835, 8837, and 8839, but not in the remaining strains. Additionally, we found missense mutations in genes encoding enzymes contributing to lipooligosaccharide (LOS) biosynthesis, such as MKAN_27485, encoding the polyketide synthase *pks5* (strains 1580, 3657, 4404, 8835, 8837, and 8839), and MKAN_27535, encoding the acyltransferase *papA4* (strains 1580, 3657, 8837, and 8839). In two other isolates (10742 and 10953), two non-synonymous mutations, one in locus MKAN_18440 (*cmaA2* – Rv0503c ortholog – c.29C > A p.Thr10Lys), and another in locus MKAN_21605 (*sigH* – Rv3223c ortholog – c.258G > T p.Met86Ile), were found.

## Discussion

In the present study, we verified the hypothesis that infection of resistant mice with highly virulent *M. kansasii* isolates from patients with lung disease, instead of the reference *M. kansasii* strain, using a high dose of bacilli for intratracheal inoculation, may result in more severe lung disease than that usually seen in the murine models of *M. kansasii* infection. We evaluated available bacterial strains from our collection of *M. kansasii* clinical isolates for virulence-associated features in the macrophage infection model to select the strains with a high degree of virulence. Based on *in vitro* results, five strains exhibiting a distinct degree of *in vitro* virulence were infected in C57BL/6 mice. The results demonstrated that highly virulent *M. kansasii* isolates, as well as ATCC strain 12478, were able to cause productive and chronically progressive infection in mice, reproducing signs of necrotic pulmonary pathology seen in human immunocompetent patients, such as caseous encapsulated granulomas, occlusion of bronchial airways with necrotic masses, and interstitial fibrosis.

Remarkably, encapsulated necrotic granulomas observed in the lungs of *M. kansasii*-infected mice in this model have not been detected in any other model of *M. kansasii* infection or the C57BL/6 model of infection with *M. tuberculosis*. Solid granulomas in the lungs are a typical pathological manifestation of experimental TB in C57BL/6 mice caused by low-dose aerosol infection of *M. tuberculosis*; and small solid granulomas were detected in mice of this lineage following intranasal infection with a high dose of *M. kansasii* (Wieland et al., 2006). The use of highly virulent clinical isolates and intratracheal route for administering mycobacteria allowed increasing severity of TB in C57BL/6 mice, with extensive necrotizing TB pneumonia (Ribeiro et al., 2014; Almeida et al., 2017; Bomfim et al., 2021). Possibly, the use of intratracheal, *trans*-laryngeal, or intranasal routes to access the respiratory pathway in small rodents increases the severity of the induced disease since these techniques, in contrast to the aerosol inhalation, do not exclude administration of small clumps of bacteria that can increase the fitness of the inoculated bacteria, despite presenting the same number of colony-forming units in the inoculated and in the inhaled dose.

The virulence of *M. kansasii* strains was a determining factor of the disease severity in our model since the low virulent strain 6849 could not cause persistent infection. Accordingly, two strains exhibiting increased virulence (8835 and 10953) induced more rapid disease progression, leading to premature death in a proportion of the infected animals. The lethality rate was one criterion for discrimination of highly virulent *M. kansasii* strains and those with an intermediate level of virulence (Supplementary Table 1).

Overall, we observed a good correlation between *M. kansasii* virulence determined with *in vitro* and *in vivo* models. Previously, *in vitro* evaluation of survival fitness and cytotoxicity of mycobacteria in macrophage culture has been shown as a good predictor for virulence of *M. tuberculosis* isolates (Park et al., 2006; Lasunskaia et al., 2010). In the early stage of infection, both *M. tuberculosis* and the phylogenetically closely related *M. kansasii* use a type VII secretion system, encoded by ESX-1 genes, to secrete essential virulence factors, such as ESAT-6 and CFP-10. These proteins are involved in the phagosomal escape of mycobacteria and regulation of macrophage viability and activation (Houben et al., 2012; Wang et al., 2015; Jankute et al., 2017; Jagielski et al., 2020), contributing to the bacterial pathogenicity. However, *in vivo, M. tuberculosis* is generally more virulent and better adapted to the human host than *M. kansasii*. Few TB bacilli compared to a high dose of *M. kansasii* is therefore needed for induction of a persistent infection in humans. However, due to the ability of *M. kansasii* to grow on low levels of nutrients and form biofilms which promote their survival and dissemination in drinking water distribution systems, household plumbing and shower heads (Falkinham, 1996), the situations when individuals receive an unusually high dose of these bacteria with aerosol might be more common than expected. Exposure to a high dose of infection with highly virulent *M. kansasii* strain may increase the probability of the disease development in the infected susceptible individuals.

We observed that all highly virulent *M. kansasii* isolates exhibited the R-colony morphotype, whereas less virulent strain 6849 displayed the S-type smooth colonies. Although a small number of *M. kansasii* strains were studied in this work, including only one S-type isolate, our results complement previous observations establishing higher clinical importance of R-type NTM strains, which are more frequently involved in severe and persistent infections compared to the S-type. It was demonstrated that in contrast to the smooth type, R-type *M. kansasii* exhibit alterations in outer membrane lipids, reducing the expression of polar, hydrophilic lipooligosaccharides (LOS), therefore increasing the proportion of less polar glycolipids, such as PDIMs, LAM, TDM, and others, which were otherwise masked or coated by LOS (Nobre et al., 2014), leading to an increase of overall hydrophobicity, virulence and probability of bacterial transmission with aerosol (Belisle and Brennan, 1989; Jankute et al., 2017). It is noteworthy that the least virulent strain 6849 was isolated from the patient with lung disease that additionally presented two comorbidities, an infection with hepatitis C virus infection and chronic cardiovascular disease, known to alter the immune system function and leading to certain level of immunodeficiency (Wu et al., 2015). The lack of clinical data from other patients with *M. kansasii* infection is a limitation of this study because it did not allow us to correlate the strain virulence with the severity of disease.

Mutations in LOS biosynthesis genes have been associated with the transformation from S- to R-colony morphology in a variety of NTM (Van der Woude et al., 2012), including *M. kansasii* (Luo et al., 2021). In the Brazilian isolates, exhibiting R-morphotype, we detected missense mutaitions in at least two genes encoding the LOS biosynthesis enzymes, MKAN_27485, which encodes the polyketide synthase *pks5* (strains 1580, 3657, 4404, 8835, 8837, and 8839), and MKAN_27535, encoding the acyltransferase *papA4* (strains 1580, 3657, 8837, and 8839). It should be yet determined whether the observed gene alterations could render loss-of-function. Additionally, since LOS expression on the bacterial surface can be altered by various factors regulating LOS-genes transactivation, lipid synthesis, degree of glycosylation, and eventual transportation to the cell surface (Burguière et al., 2005), it is crucial to establish the cell wall lipid content and composition of virulence-associated proteins secreted during *M. kansasii* infection. How the temporal lipid and protein profiles determine the fate of infection remains to be clarified, and a more significant number of smooth variants need to be compared with the rough variants for this purpose.

We observed that VF genes belonging to the functional category “virulence, detoxification, adaptation” have undergone a massive reduction in *M. kansasii* when compared to *M. tuberculosis* H37Rv, including the significant decrease in the toxin–antitoxin’s subsystem genes previously reported by Wang et al. (2015). Additionally, in accordance with previous observations (Wang et al., 2015; Tagini et al., 2021), our analysis showed the presence of all key RD1 genes, including the ESAT-6 and CFP-10, coding for the essential components of the ESX-1 secretion system, and the loci encoding for the ESX-1 secretion-associated proteins, ESP. These genes were conserved in all genomes of the Brazilian *M. kansasii* isolates, although some of the isolates exhibited heterogeneity in copy number (data not shown) and numerous SNPs in these genes. However, we observed identical SNPs in gene MKAN_07615, encoding for ESX secretion-associated protein EspG, in several geographically distant isolates, such as Pernambuco (strains 8835, 8837, and 8839) and Rio de Janeiro (strains 1580, 3657, and 4404) (Machado et al., 2018). Besides the possible influence of presence, absence, or copy number of specific genes, the presence of SNPs can also interfere with virulence phenotype. Accordingly, as the Rv0503c and Rv3223c genes, encoding cmaA2 and sigH virulence factors, respectively, have a known impact on the virulence of *M. tuberculosis*, we can hypothesize that mutations observed in the respective orthologs MKAN_18440 (*cmaA2*) and MKAN_21605 (*sigH*) might have an impact on the virulence profile of the strain 10953, since only this isolate carry non-synonymous mutations in these loci. However, transfection experiments are needed to provide experimental evidence for the actual contribution of the proposed genetic elements for the virulence of *M. kansasii*.

Comparing the genome sequences of the *M. kansasii* isolates investigated in the present work (Machado et al., 2018), we demonstrated that the most virulent (8835) and the less virulent (6849) strains display a high level of genetic difference compared to the reference strain ATCC 12478 (more than 10,000 SNPs). On the other hand, three virulent strains isolated from patients in Rio de Janeiro are genetically highly similar to the strain 12478 (less than 100 SNP of difference), isolated 70 years ago in Kansas (United States). Previous genotyping studies of *M. kansasii* isolates, based on pulsed-field gel electrophoresis and multi-locus sequence typing (Ross et al., 1992; Alcaide et al., 1997), as well as recent phylogenomic analyses of more than two hundred genomes of a worldwide collection of *M. kansasii* isolates, including Brazilian isolates (Luo et al., 2021; our unpublished data, manuscript in preparation), revealed a close-relatedness between the strain ATCC 12478 and subtype I *M. kansasii* strains isolated from patients in European countries and Americas, suggesting both clonality of specific genotypes and wide geographic distribution of the most prevalent subtype found in humans. These data strongly suggest that a few clones sharing several pathogenic features have spread widely within the *M. kansasii* population, probably reflecting an evolvement of a new lung pathogen from an environmental waterborne organism. Further evolution of the local strains in each geographic region and widespread increase of susceptible aged population contribute to a further selection of more virulent variants, as has been observed in *M. tuberculosis* (Stucki et al., 2016).

In conclusion, we propose the infection of resistant C57BL/6 mice with *M. kansasii* as a reliable model reproducing human-like necrotic lung pathology, therefore suitable for investigating *M. kansasii* virulence and pathogenicity, as well as an anti-mycobacterial or adjunct drug testing. Additionally, a macrophage infection model *in vitro* may be used to predict the virulence of *M. kansasii* strains. Significant heterogeneity in virulence of Brazilian *M. kansasii* clinical isolates was demonstrated, with a high prevalence of strains displaying high or intermediate virulence. The genomic similarity of some strains isolated from patients in different geographic regions of the world, including Brazil, suggests that some pathogenic *M. kansasii* variants might be globally distributed.

## Data Availability Statement

The datasets presented in this study can be found online at https://www.ncbi.nlm.nih.gov/genbank/, under the following accession numbers: PQOQ00000000, PQOS00000000, PQOT00000000, PQOU00000000, PQOP00000000, PQOL00000-000, PQON00000000, PQOR00000000, PQOV00000000, and PQOW00000000.

## Ethics Statement

The animal study was reviewed and approved by Animal Care and Use Committee of State University of North Fluminense (Permit number 350/2017).

## Author Contributions

EL, VM, and PS designed and conceived the experiments. VM, TS, FA, SC, GS, SV, and EM performed the experiments. LC identified mycobacterial isolates. EL, VM, TS, PS, EM, and MC analyzed the data and wrote the manuscript. EC and MC contributed with reagents, materials, and analysis tools. VM, TS, FA, EM, PS, and EL have made substantial contributions to the work and approved its final version. All authors contributed to the article and approved the submitted version.

## Conflict of Interest

The authors declare that the research was conducted in the absence of any commercial or financial relationships that could be construed as a potential conflict of interest.

## Publisher’s Note

All claims expressed in this article are solely those of the authors and do not necessarily represent those of their affiliated organizations, or those of the publisher, the editors and the reviewers. Any product that may be evaluated in this article, or claim that may be made by its manufacturer, is not guaranteed or endorsed by the publisher.

## Footnotes

1
https://www.ncbi.nlm.nih.gov/nuccore/CP006835.1

## Supplementary Material

Supplementary Figure 1 Virulence-associated properties of *M. kansasii* strains evaluated in the macrophage-based model of infection. RAW 264.7 macrophage-like cells were infected with different strains and incubated for 4 days. **(A)** The numbers of CFU recovered from macrophages infected at a MOI of 1:1 were quantified at days 1 and 4 p.i. The bacterial growth rate is presented as mean ± SD of increases in the log_10_ numbers of intracellular CFU measured in two separate experiments. **(B)** Induction of necrotic cell death in the cell cultures infected at a MOI of 10:1. LDH release from the dead cells was measured in the culture supernatants at day 3 p.i., and the percentage of cytotoxicity was calculated as described in “Materials and Methods” section. Asterisks indicate the isolates for which the values of growth or cytotoxicity differed significantly from the respective value of the reference ATCC 12478 strain; **P* < 0.05; ***P* < 0.01; ****P* < 0.001.

Supplementary Table 1 Relative virulence of *Mycobacterium kansasii* clinical isolates in comparison with the reference ATCC strain 12478 evaluated in the *in vitro* model of infected macrophages and in the *in vivo* model of C57BL/6 mice infection.

Supplementary Table 2 457 *Mycobacterium tuberculosis* H37Rv virulence factors loci and their orthologs in *Mycobacterium kansasii* ATCC 12478 strain.

Supplementary Table 3 Single nucleotide polymorphisms in virulence factors genes identified in 12 *Mycobacterium kansasii* strains compared to the reference strain *Mycobacterium kansasii* ATCC 12478.
