# Supplementary figures and images for: A Murine Model of Mycobacterium kansasii Infection Reproducing Necrotic Lung Pathology Reveals Considerable Heterogeneity in Virulence of Clinical Isolates
Source: Front Microbiol. 2021 Aug 24;12:718477. doi: 10.3389/fmicb.2021.718477 (PMC8422904; doi:10.3389/fmicb.2021.718477)

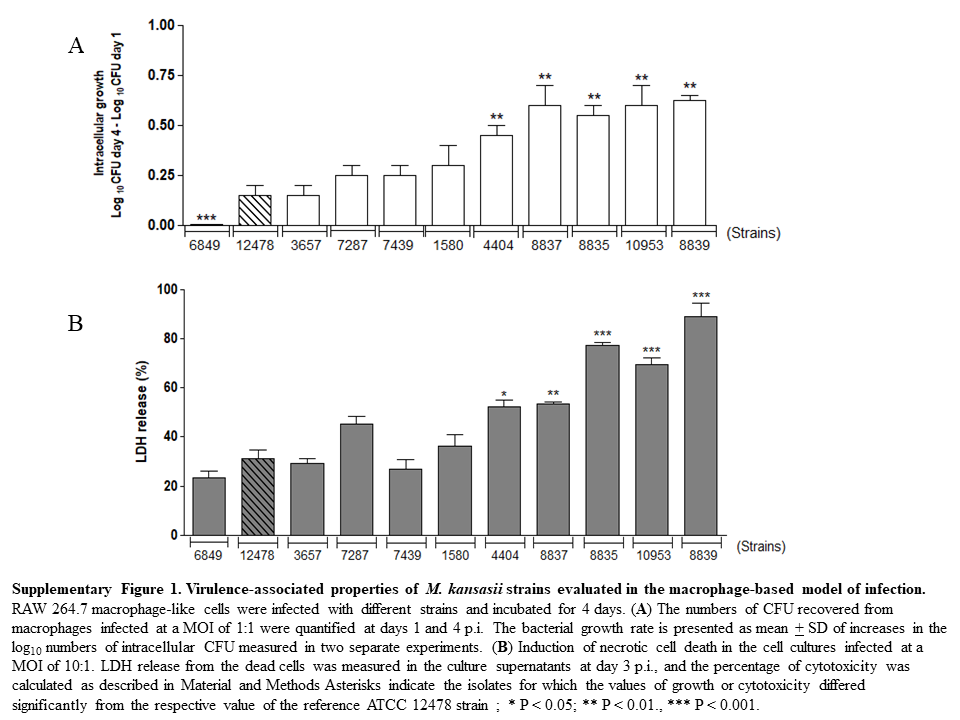

Supplement: Supplementary Figure 1 — Virulence-associated properties of M. kansasii strains evaluated in the macrophage-based model of infection. RAW 264.7 macrophage-like cells were infected with different strains and incubated for 4 days. (A) The numbers of CFU recovered from macrophages infected at a MOI of 1:1 were quantified at days 1 and 4 p.i. The bacterial growth rate is presented as mean ± SD of increases in the log10 numbers of intracellular CFU measured in two separate experiments. (B) Induction of necrotic cell death in the cell cultures infected at a MOI of 10:1. LDH release from the dead cells was measured in the culture supernatants at day 3 p.i., and the percentage of cytotoxicity was calculated as described in “Materials and Methods” section. Asterisks indicate the isolates for which the values of growth or cytotoxicity differed significantly from the respective value of the reference ATCC 12478 strain; *P < 0.05; **P < 0.01; ***P < 0.001. [file Image_1.TIF]
