# Supplementary material for: A Murine Model of Mycobacterium kansasii Infection Reproducing Necrotic Lung Pathology Reveals Considerable Heterogeneity in Virulence of Clinical Isolates
Source: Front Microbiol. 2021 Aug 24;12:718477. doi: 10.3389/fmicb.2021.718477 (PMC8422904; doi:10.3389/fmicb.2021.718477)
Supplement: Supplementary Table 1 — Relative virulence of Mycobacterium kansasii clinical isolates in comparison with the reference ATCC strain 12478 evaluated in the in vitro model of infected macrophages and in the in vivo model of C57BL/6 mice infection. [file Table_1.DOCX]

**Supplementary Table 1. Relative virulence of *M. kansasii* clinical isolates in comparison with the reference ATCC strain 12478 evaluated in the *in vitro* model of infected macrophages and in the *in vivo* model of C57BL/6 mice infection.**

| **Strain** | **Relative value of intracellular growth in macrophages**  **(Index A)** | **Relative value of necrotic macrophage death induction**  **(Index B)** | **Relative virulence index**  **[(Index A + Index B)/2]** | **Virulence Pattern ***  ***In vitro*** | **Virulence Pattern***  ***In vivo*** |
| --- | --- | --- | --- | --- | --- |
|  |  |  |  |  |  |
|  |  |  |  |  |  |
| Reference strain  12478 | 1 | 1 | 1 | II | II |
| 1580 | 2 | 1.17 | 1.59 | II | not tested |
| 3657 | 1 | 0.95 | 0.98 | II | not tested |
| 4404 | 3 | 1.68 | 2.34 | II | II |
| 7287 | 1.67 | 1.46 | 1.57 | II | not tested |
| 7439 | 1.67 | 0.86 | 1.27 | II | not tested |
| 6849 | 0.1 | 0.75 | 0.4 | III | III |
| 8835 | 3.6 | 2.5 | 3.09 | I | I |
| 8837 | 4 | 1.72 | 2.86 | I | not tested |
| 8839 | 4.2 | 2.87 | 3.56 | I | not tested |
| 10953 | 4 | 2.25 | 3.13 | I | I |

***Pattern I** (increased virulence) – relative virulence index in testing *in vitro-* more than 2.5; when applied to *in vivo* testing: chronic productive infection, necrotic lung pathology and/or premature animal death;

**Pattern II** (intermediate virulence) –relative virulence index in testing *in vitro-* 0.41-2.5; when applied to *in vivo* testing: chronic productive infection, necrotic lung pathology, animal survival up to 150 dpi;

**Pattern III** (low virulence) - relative virulence rate in testing *in vitro-* 0.4 or less; when applied to *in vivo* testing: non-persistent infection, elimination of bacteria from lungs.
